# Relationship Between Oral Health and Clinical Osteoporosis Among Hospitalized Patients with and Without Diabetes

**DOI:** 10.7759/cureus.7145

**Published:** 2020-02-29

**Authors:** Kenneth Izuora, Gayle Allenback, Amber Champion, Civon Gewelber, Michael Neubauer

**Affiliations:** 1 Internal Medicine/Endocrinology, University of Nevada, Las Vegas, USA; 2 Biostatistics, Independent Biostatistician, Las Vegas, USA; 3 School of Dental Medicine, University of Nevada, Las Vegas, USA

**Keywords:** oral health, osteoporosis, diabetes mellitus

## Abstract

Objective

Diabetes mellitus (DM) is associated with poor oral health and osteoporosis (OP). The aim of this study was to assess the relationship between OP, periodontal disease (PD), and other dental and health outcomes in a cohort of hospitalized patients with and without DM.

Method

Using a cross-sectional study design, we enrolled consecutive hospitalized patients. We administered a questionnaire to gather demographic information, oral health history, smoking history, and history of OP. We inspected their dentition and reviewed their charts. Data were analyzed using t-tests, chi-square tests, and logistic regression models.

Result

Out of 301 patients enrolled, 275 had PD, 102 had DM, and 30 had OP. In univariate analyses, factors associated with OP included older age (p<0.001), female gender (p=0.046), presence of DM (p=0.049), and having more discharge medications (p=0.01). There was no significant relationship between PD and OP. In logistic regression analyses, age remained significantly associated with having OP among all hospitalized patients and in the non-DM populations. In the DM population, female gender was the only significant predictor for having OP.

Conclusion

Although we found no significant relationship between having PD and OP in our population, we found that among patients with DM, female gender predicted OP, whereas in patients without DM, age was a stronger predictor. Earlier screening for OP in female patients with DM may be useful in identifying and treating OP sooner in this population.

## Introduction

Diabetes mellitus (DM) is a known risk factor for osteoporosis (OP) and is also associated with a higher prevalence of periodontal disease (PD) [1-3]. PD is associated with poor oral health and results in tooth loss and increased chronic systemic inflammation [4-8]. Inflammation, in addition to being associated with poorer glycemic control and increased risk for cardiovascular disease, is also associated with OP and increased fracture risk [9-12]. Furthermore, there is a known association between OP and PD [13-16]. PD, OP, and DM all share common risk factors like older age, inflammation, tobacco use, and stress but could also directly interact with one another. To explore the relationship between PD, OP, and DM further, we analyzed the prevalence of OP and the association of OP with oral health outcomes including PD among hospitalized patients with and without DM. 

## Materials and methods

Study design

This was a cross-sectional study. A daily census of all patients admitted at an academic medical center located in an urban center was obtained. Using convenience sampling, consecutive adult patients, independent of the reason for their hospitalization, were approached for enrollment. The most common reasons for hospitalization included chest pain, abdominal pain, shortness of breath (chronic obstructive pulmonary disease and congestive heart failure exacerbation), syncope, and diabetic foot ulcers. Following an explanation of the purpose of the study, informed consent was obtained from eligible patients who agreed to participate. We included all adult non-pregnant patients (age >18 and <89 years) hospitalized for at least one day who were able to understand and consent to the study. Patients with altered mental status, terminal illness, or those admitted in the intensive care unit were excluded. For the DM population, a minimum duration of DM of one year was required for enrollment. This was done to exclude those who were newly diagnosed and still undergoing adjustment of their DM therapy. The study was approved by the hospital Institutional Review Board.

Study procedures

Participants were interviewed using an investigator-administered questionnaire to gather information on their demographics, oral health history, smoking history, and medical history (including screening for OP). Their oral cavity was inspected to count the total number of their remaining teeth and to assess for broken or decayed teeth. We did not perform any invasive dental procedures. Medical information obtained was validated through a review of individual medical records.

Before starting enrollment, the study team met and reviewed several images of teeth in various degrees of health obtained from our dental clinic. There was agreement between all team members on the health and number of teeth in the samples reviewed. This was done to calibrate the findings reported by each team member during the enrollment of participants for consistency.

The inspection of the oral cavity was conducted using a tongue depressor, mouth mirror, and a flashlight. All the natural teeth were counted and then, out of these, the number of teeth that were broken or decaying was determined. The number of healthy teeth was determined by subtracting the number of decayed or broken teeth from the total number of teeth.

Responses on the questionnaire that were considered to suggest the presence of PD (i.e., gingivitis or periodontitis) were based on a validated self-reported survey tool [17]. These responses included any positive answer to questions about past history of gum or PD, prior deep cleaning, loose teeth, tooth sensitivity, or gum bleeding during brushing.

We obtained information about dental hygiene practices by asking for the number of times a week that the participants brushed, flossed, or used mouthwash.

The presence of clinical OP was determined based on one of the following prespecified criteria: prior diagnosis with OP, current treatment for OP, or history of low trauma fracture after age 40 years.

Patients with history of fractures resulting from significant trauma like motor vehicle accidents or during contact sports were not assessed to have OP in this study. Also, fractures occurring before age 40 years were not included as OP-related fractures in this study.

Statistical analysis

Statistical analyses were performed using SPSS v25 (IBM, Armonk, NY). Continuous variables (such as age) were described with means and standard deviations (as tests of skewness and kurtosis demonstrated no significant variation from a normal distribution), and categorical variables (such as gender) were described with counts and proportions. Univariate analyses included independent sample t-tests and chi-squares/Fisher’s exact. Multivariate analyses included logistic regression, where variables either significant in the univariate analyses or deemed clinically relevant with respect to OP were entered into the model for the particular patient population in question. A p-value of <0.05 was considered to indicate statistical significance.

## Results

A total of 301 patients were enrolled (129 female and 172 male). Out of this population, 102 participants (34%) had DM and 199 (66%) did not have DM. Participants with DM were older (55.8 vs. 48.1 years, p<0.001), had fewer healthy teeth (14.2 vs. 20.5 teeth, p<0.001), and had greater body mass index (31.8 vs. 27.3 kg/m^2^, p<0.001). Overall, 275 participants (91%) had PD, whereas 30 participants (10%) had OP (Table 1).

**Table 1:** Description of study population DM, diabetes mellitus.

	No DM (n=199)	DM (n=102)	P-value
Age (years)	48.1 years (15.5 SD)	55.8 years (11.9 SD)	<0.001
Number of healthy teeth	20.5 (8.9 SD)	14.2 (9.8 SD)	<0.001
Body mass index (kg/m^2^)	27.3 (6.9 SD)	31.8 (7.3 SD)	<0.001
Gender			
Female	85 (42.7%)	44 (43.1%)	0.944
Male	114 (57.3%)	58 (56.9%)	0.944
Ethnicity			
White	94 (47.2%)	42 (41.2%)	0.317
Hispanic or Latino	29 (14.6%)	18 (17.6%)	0.487
Black or African American	55 (27.6%)	29 (28.4%)	0.885
Asian	8 (4.0%)	5 (4.9%)	0.768
American Indian/Alaska Native	0 (0.0%)	1 (1.0%)	0.339
Native Hawaiian/Pacific Islander	2 (1.0%)	3 (2.9%)	0.341
Other	11 (5.55%)	4 (3.9%)	0.544
Periodontal disease			
Yes	179 (89.9%)	96 (94.1%)	0.223
No	20 (10.1%)	6 (5.9%)	0.223
Osteoporosis			
Yes	15 (7.5%)	15 (14.7%)	0.049
No	184 (92.5%)	87 (85.3%)	0.049
Smoking history			
Yes	123 (62.1%)	67 (66.3%)	0.474
No	75 (37.9%)	34 (33.7%)	0.474

In univariate analysis, the factors that were significantly associated with the presence of OP included older age (p<0.001), female gender (p=0.046), diagnosis of DM (p=0.049), and having a greater number of discharge medications (p=0.01). Diagnosis with PD, positive smoking history, or the number of healthy teeth of the participants were not associated with OP (p=1, 0.439, and 0.436, respectively, Table 2).

**Table 2:** Univariate analyses for osteoporosis presence OP, osteoporosis; DM, diabetes mellitus; PD, periodontal disease.

	Patients with OP	Patients without OP	P-value
Age (mean)	58.73 (SD 10.11)	49.82 (SD 15.01)	<0.001
Female gender (DM patients only)	66.7%	39.1%	0.046
Number of discharge medications (mean)	7.1 (SD 4.38)	5.23 (SD 3.62)	0.010
DM diagnosis	50.0%	32.1%	0.049
PD diagnosis	93.3%	91.1%	1.000
Smoking history	70.0%	62.8%	0.439
Number of healthy teeth (mean)	17.00 (SD 8.07)	18.46 (SD 9.82)	0.436

In logistic regression analyses that included the variables that were associated with OP either statistically in the univariate analyses (Table 2) or clinically (PD and smoking), age remained a significant predictor of OP in the overall patient population (ExpB=1.044, 95% CI: 1.013-1.076; p=0.006) (Table 3). Subgroup analysis according to the presence of DM revealed that, in the non-DM group, age was the only significant predictor of OP (ExpB=1.066, 95% CI: 1.023-1.110; p=0.002), after adjusting for gender (Table 4), whereas in the DM population, female gender was the only significant predictor of the presence of OP (Exp B=3.231, 95% CI: 1.003-10.408; p=0.049) (Table 5). Age itself did not significantly predict the presence of OP in the DM population, after adjusting for gender.

**Table 3:** Logistic regression model of predictors of the presence of osteoporosis in the overall population

Covariate	N	Odds ratio	95% confidence interval	P-value
Age	299	1.044	(1.013, 1.076)	0.006
Gender (female)	128	1.996	(0.914, 4.360)	0.083
Periodontal disease (present)	273	1.004	(0.214, 4.706)	0.996
Diabetes (present)	101	1.681	(0.767, 3.682)	0.194
Smoking history (positive)	190	1.241	(0.534, 2.884)	0.616

**Table 4:** Logistic regression model of predictors of the presence of osteoporosis in the non-DM population DM, diabetes mellitus.

Covariate	N	Odds ratio	95% Confidence interval	P-value
Age	198	1.066	(1.023, 1.110)	0.002
Gender (female)	84	1.271	(0.420, 3.842)	0.671
Periodontal disease (present)	178	0.424	(0.078, 2.298)	0.320
Smoking history (positive)	123	0.875	(0.269, 2.853)	0.825

**Table 5:** Logistic regression model of the predictors of the presence of osteoporosis in the DM population DM, diabetes mellitus. *All 15 DM patients who had osteoporosis also had periodontal disease.

Covariate	N	Odds ratio	95% confidence interval	P-value
Age	101	1.016	(0.968, 1.066)	0.524
Gender (female)	44	3.231	(1.003, 10.408)	0.049
Periodontal disease* (present)	95	318731766.425	(0.000, .)	0.999
Smoking history (positive)	67	1.487	(0.422, 5.236)	0.536

## Discussion

In this hospital-based study, we found that several of the traditional predictors of OP (age, female gender, and having DM) were associated with a diagnosis of OP in our population. We did not find significant association between having PD and OP. When accounting for the other variables in multivariate models, age predicted OP in the entire population and in patients without DM, whereas among patients with DM, female gender was the only significant predictor of OP.

In addition to the traditional risk factors associated with OP, several reports have suggested that there was a significant association between PD and OP. Kalinowski et al. found a higher Fracture Risk Assessment Tool (FRAX) probability for fracture among patients with more severe periodontitis among men and women aged 40-89 years [18]. Another study by Richa et al. found increased clinical attachment loss, bleeding, and gingivitis (all measures of PD) among postmenopausal women with OP compared to those without OP [15]. In a study examining PD in DM patients, deMiguel-Infante et al. found significant associations between missing teeth, OP, and a higher risk for PD [16]. Unlike these studies, our study did not find an association between PD and having a diagnosis of OP independent of the presence of DM. A possible explanation for this finding could be because of the low prevalence of OP in our population that had a younger age when compared to the typical age for patients diagnosed with OP.

In subgroup analyses, we found no difference in gender distribution between the DM and non-DM populations (Table 1). However, in multivariate analyses, unlike in the general and non-DM populations (Tables 3, 4), female gender (not age) predicted OP among DM patients (Table 5). This suggests that among female patients who are already at a higher risk for developing OP, those with DM had a greater risk of OP-related bone loss compared to those female patients without DM after taking into account their age. The clinical relevance of these findings is that earlier OP screening should be considered for female patients with DM who are similar to our population than is recommended according to current general OP screening guidelines (Table 1).

## Conclusions

Contrary to expected results, we found that having PD was not significantly associated with clinical OP among our population consisting of hospitalized patients with and without DM. This finding may be related to the low prevalence of clinical OP in our study population.

The usual predictors for OP were associated with the presence of clinical OP in our population. However, among patients with DM, female gender and not age predicted OP. This suggests that female patients with DM similar to the population in this study may be at the highest risk for developing OP; hence, earlier screening may be useful in promptly identifying and treating OP in this population.
